# Colonic Inflammation in a Samoan Immigrant with Gastric Lymphoma Shown by Positron Emission Tomography

**DOI:** 10.4269/ajtmh.14-0464

**Published:** 2015-05-06

**Authors:** Ashley Burt, Carl Hoh

**Affiliations:** Department of Radiology, University of California at San Diego Medical Center, San Diego, California

**Figure 1.**
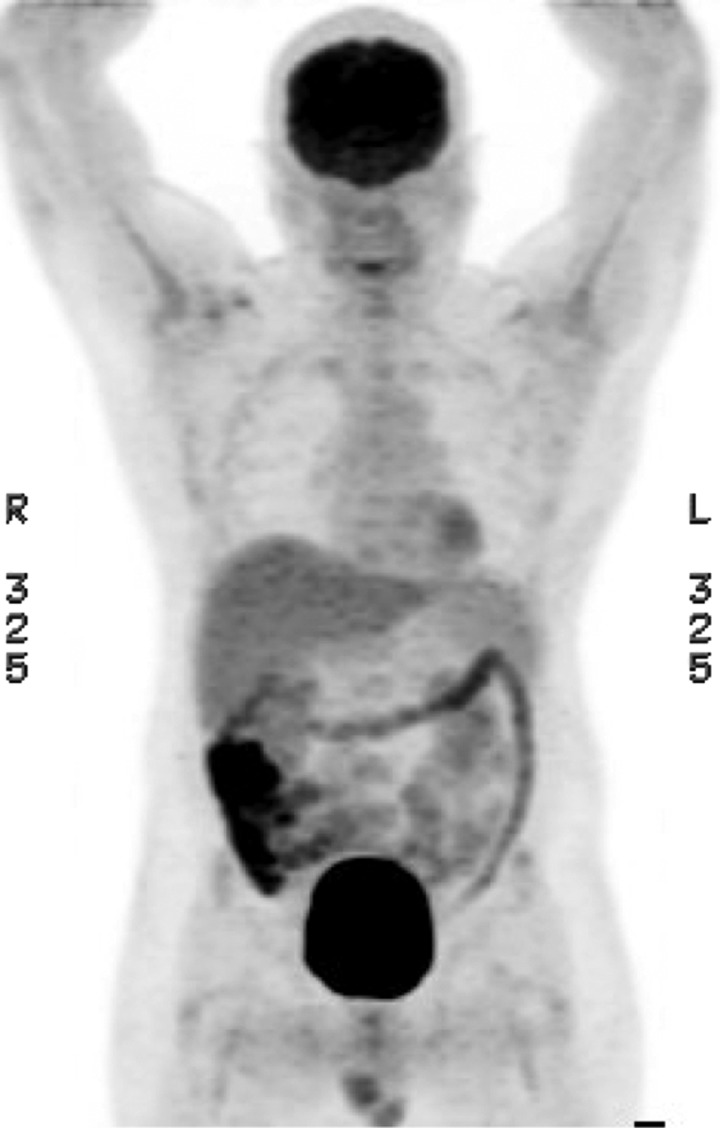
PET/CT 1-10-2013.

**Figure 2.**
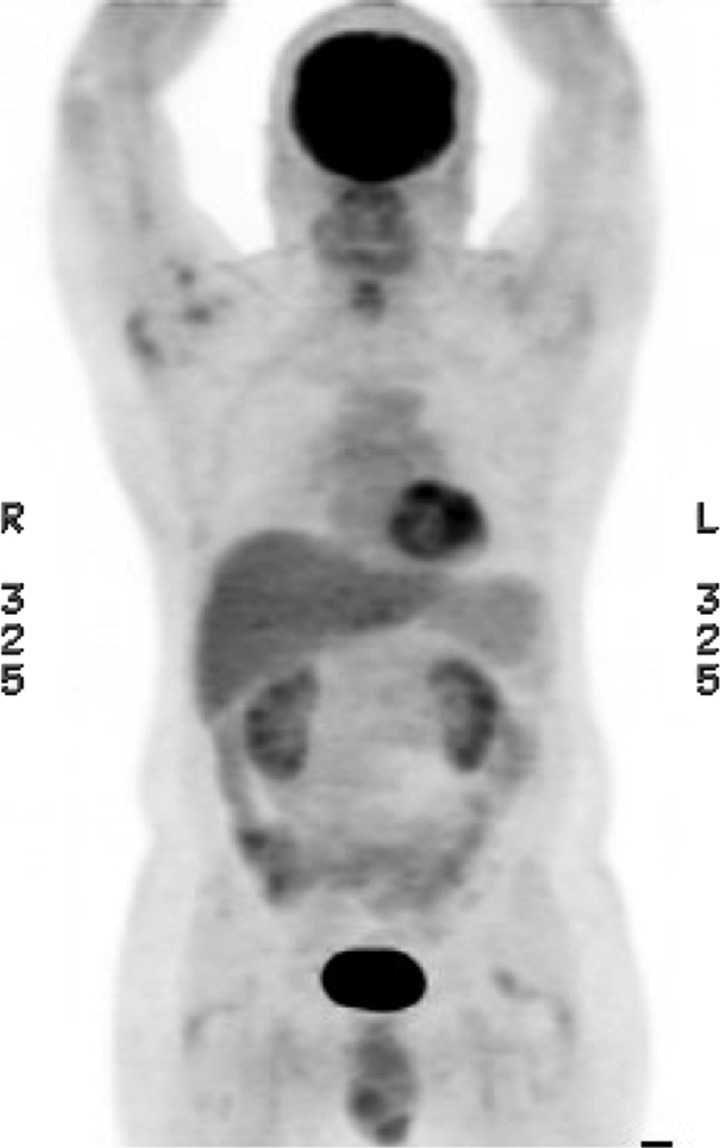
PET/CT 4-05-2013.

A 57-year-old Samoan man with large B-cell gastric lymphoma presented with fatigue, anemia, and melena. A positron emission tomography (PET) with 18-fluoro-2-deoxyglucose (FDG) showed no gastric activity but marked hypermetabolic activity in the cecum and ascending colon (Figure 1

). Corresponding computed tomography (CT) revealed bowel wall thickening, consistent with infectious or inflammatory process. Colonoscopy showed non-specific inflammatory changes. Laboratory work showed leukocytosis (white blood cells = 19.3) and eosinophilia (absolute eosinophil count [AEC] = 9.2). Extensive infectious disease workup was only significant for a positive *Strongyloides* antibody (3.11; normal < 1.5). He received two doses of ivermectin 2 weeks apart.^1^ Post-treatment PET/CT showed resolution of FDG uptake and normalization of bowel wall thickness (Figure 2

). Concurrent laboratory tests confirmed resolution of the infection with negative antibodies (0.35).

*S. sterocoralis* nematodes typically inhabit the small bowel; therefore, involvement of the cecum and ascending colon in this patient suggests a hyperinfection syndrome. Diagnosis of *Strongyloides* may be established by detection of larvae in stool or a biopsy or by serology. PET and CT are not typically used for diagnosis. However, this case shows *Strongyloides* hyperinfection syndrome by PET as an incidental finding and reinforces the need to maintain a high index of suspicion for this infection in immigrants from endemic regions.
